# A promising area of research in medicine: recent advances in properties and applications of Lactobacillus-derived exosomes

**DOI:** 10.3389/fmicb.2024.1266510

**Published:** 2024-04-15

**Authors:** Rui Liu

**Affiliations:** School of Food Engineering, Ludong University, Yantai, Shandong, China

**Keywords:** Lactobacillus-derived exosomes, isolation methods, immunomodulation, intestinal microecological balance, genetic engineering

## Abstract

Lactobacillus-derived exosomes, small extracellular vesicles released by bacteria, have emerged as a promising area of research in recent years. These exosomes possess a unique structural and functional diversity that allows them to regulate the immune response and promote gut health. The isolation and purification of these exosomes are crucial for their effective use as a therapeutic agent. Several isolation and purification methods have been developed, including differential ultracentrifugation, density gradient centrifugation, and size-exclusion chromatography. Lactobacillus-derived exosomes have been demonstrated to have therapeutic potential in various diseases, such as inflammatory bowel disease, liver disease, and neurological disorders. Moreover, they have been shown to serve as effective carriers for drug delivery. Genetic engineering of these exosomes has also shown promise in enhancing their therapeutic potential. Overall, Lactobacillus-derived exosomes represent a promising area of research for the development of novel therapeutics for immunomodulation, gut health, and drug delivery.

## Introduction

Lactobacilli are a group of Gram-positive bacteria widely distributed both within and outside of the human body, which produce lactic acid by fermenting lactose and other carbohydrates (Bintsis, 2018; Ibrahim and Ouwehand, 2019). They have been extensively studied and applied as a crucial probiotic in the food industry, animal husbandry, and healthcare (Alayande et al., 2020; Ayivi et al., 2020). Their probiotic effects include promoting intestinal health, enhancing immunity, and regulating the balance of intestinal flora (Mitsuoka, 2000; Tsai et al., 2012; Deng et al., 2022). Moreover, exosomes secreted by lactic acid bacteria have gained increasing attention as a novel type of biological agent (González-Lozano et al., 2022).

Exosomes are small extracellular vesicles secreted by cells via the endocrine pathway, ranging from 20 to 400 nm in size, and containing a variety of bioactive substances such as proteins, nucleic acids, and metabolites (Yu et al., 2023). They are widely produced in various organisms such as bacteria, fungi, plants, and animals (Schuh et al., 2019; Zhou et al., 2022), and are involved in multiple biological processes, including cell signaling, pathogenic microorganism infection, and immune regulation (Wang et al., 2017; Li et al., 2019). As their importance is gradually recognized, their applications in medicine, agriculture, and other fields are gaining attention. Currently, the study of exosomes has become one of the hotspots in the field of biology.

In recent years, the study of Lactobacillus-derived exosomes has attracted widespread attention, as they have been shown to possess various biological functions, such as antibacterial (Lee et al., 2021), immunomodulatory (Liu et al., 2021b), and intestinal protection (Choi et al., 2020; Tong et al., 2021). Recent studies have revealed that the protein, DNA, and RNA components in Lactobacillus-derived exosomes can exert a broad range of effects on the host through the transfer of commensal microorganisms in the intestine (Liu et al., 2021a). Additionally, the components and mechanisms of action of Lactobacillus-derived exosomes are gradually being revealed. For instance, Lactobacillus-derived exosomes can modulate the host immune system, improve the imbalance of intestinal flora, and inhibit the growth of harmful bacteria, thus having a wide range of applications in food, healthcare, and agriculture. In this paper, we will review the recent advances in the properties and functions of Lactobacilli exosomes, isolation and preparation methods, engineering modifications, and their applications.

## Structure and function of Lactobacillus-derived exosomes

### Structure

Similar to the structure of exosomes from other sources, Lactobacillus-derived exosomes are extracellular vesicles that are secreted by Lactobacillus and have a diameter between 20 and 400 nm, usually consist of one or more lipid bilayers, wrapped in a similar structure to the cell membrane (Yu et al., 2019; Gu et al., 2021). These exosomes contain a variety of bioactive components such as proteins, polysaccharides, and lipids. Lactobacillus exosome proteins are important components and consist of a variety of enzymes and structural proteins (Behzadi et al., 2017; Domínguez Rubio et al., 2017). These enzymes play a vital role in breaking down food and regulating flora in the intestine. Polysaccharides in exosomes are also essential components that promote the proliferation and differentiation of intestinal mucosal cells and enhance intestinal immunity (Huang et al., 2017; Sha et al., 2021). Nucleic acids in exosomes have important biological functions as RNA carriers that can transfer information and influence gene expression in the host (Munir et al., 2020; Askenase, 2021).

The complex composition and structure of Lactobacillus-derived exosomes give them diverse biological functions and wide-ranging application prospects. Current research on Lactobacillus-derived exosomes is still ongoing, and more discoveries and applications are expected to emerge in the future.

### Immunomodulation

**Figure 1:**
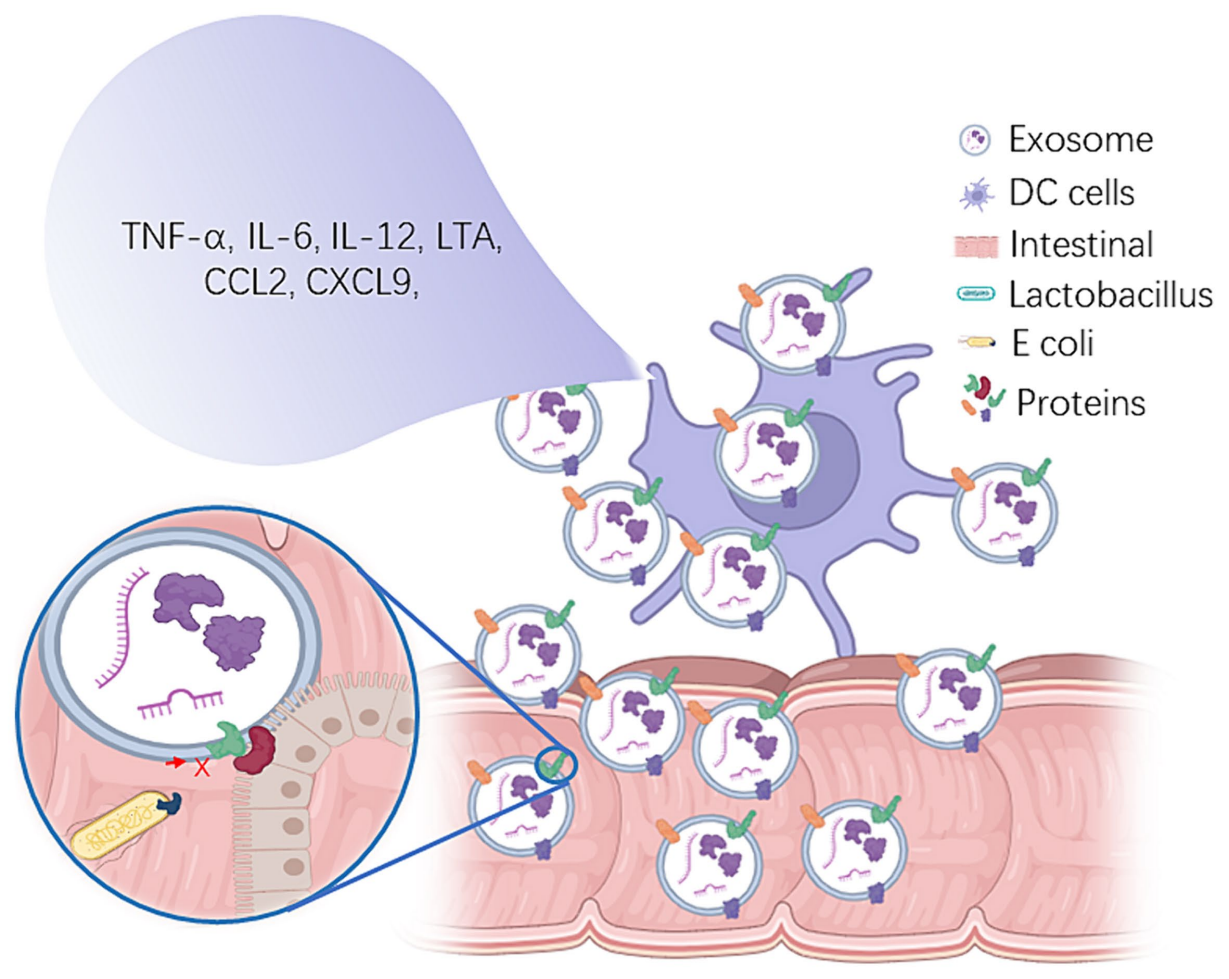
The immunomodulatory role of Lactobacillus-derived exosomes and their impact on intestinal health. Lactobacillus-derived exosomes play a pivotal role in modulating immune responses by containing proteins such as FABP6, EPCAM, and various polysaccharide molecules (Górska et al., 2014). These exosome components have the capacity to stimulate dendritic cells, resulting in the production of pro-inflammatory cytokines, including TNF-α, IL-6, IL-12, CCL2, and CXCL9 (Liu et al., 2021a,b), ultimately contributing to the potential stimulation of T cell proliferation *in vitro*. This underscores their profound influence on immune cell function and response, particularly within the context of intestinal immune regulation. Furthermore, proteins within Lactobacillus-derived exosomes exhibit a competitive binding capacity to the intestinal surface, thereby reducing the adhesion of harmful bacteria and mitigating the ensuing intestinal inflammatory response (Doron et al., 2005). Additionally, these exosomes encapsulate bioactive substances, such as extracellular enzymes, antibiotics, antimicrobials, antibiotic enzymes, iron carrier proteins, β-glucan, siRNA and polysaccharides, which collectively exert regulatory control over the intestinal microbiome (Uddin et al., 2020; Díaz-Garrido et al., 2021; Yang F. et al., 2022; Yang Q. et al., 2022; Yang Z. et al., 2022). This regulation impacts both the composition and abundance of intestinal flora, facilitating the enhancement of probiotic growth and metabolic activity within the intestinal environment, thus contributing to the maintenance of intestinal health.

Exosomes released by lactic acid bacteria have gained considerable interest due to their potential immunomodulatory effects. In particular, exosomes from Lactobacillus species have been found to exert anti-inflammatory, antibacterial, antiviral, and immune-enhancing effects (Hsu et al., 2018; Lee et al., 2018; Kang et al., 2020). These bioactive molecules are involved in several mechanisms that modulate the host immune system (Figure 1).

Lactobacillus-derived exosomes are key in modulating immune responses, primarily by activating cells like macrophages, dendritic cells, T cells, and B cells (Leung et al., 2006; Chen et al., 2008; Kim et al., 2020). These exosomes contain proteins such as Fatty Acid Binding Protein 6 (FABP6), Epithelial Cell Adhesion Molecule (EPCAM), and C1q and Tumor Necrosis Factor (TNF) related 3 (C1QTNF3), and engage in Peroxisome Proliferator Activated Receptor (PPAR) [Integrin Linked Kinase (ILK)/FABP6] pathways, alongside various polysaccharide molecules (Tang et al., 2015). These components induce dendritic cells to produce pro-inflammatory cytokines, including TNF-α, Interleukin (IL)-6, IL-12, lymphotoxin α (LTA), C-C Motif Ligand 2 (CCL2), and C-X-C Motif Ligands 9 and 10 (CXCL9 and CXCL10) (Liu et al., 2021b). Moreover, polysaccharide molecules in the exosomes trigger signal transduction in immune cells, further promoting cytokine production via immune receptor binding. The impact of these exosomes on bone marrow-derived dendritic cells (BM-DC) varies, particularly in response to *Lactoplantibacillus plantarum* WCFS1. For example, exposure to the L900/2 strain increases IL-10 production, whereas L900/3 boosts IL-12p70 production. *L. plantarum* exopolysaccharide amplifies nitric oxide, IL-12p70, and RANTES (Regulated upon Activation, Normal T Cell Expressed and Presumably Secreted; CCL5) production, while reducing IL-10 secretion in serum, intestinal fluid, and dendritic cell supernatants (Górska et al., 2014). Furthermore, this exopolysaccharide upregulates Major Histocompatibility Complex (MHC) II and Cluster of Differentiation (CD) 86 expression on DC-surfaces and stimulates T cell proliferation *in vitro*, illustrating its profound effect on immune cell function and response (Tang et al., 2015).

Recent studies reveal that lncRNAs and lincRNAs, particularly in response to Lipopolysaccharides (LPS) stimulation, play a pivotal role in immune regulation. These include lnc-IL7R, which represses proinflammatory mediators by maintaining a repressive chromatin mark, and NeST Long noncoding (lnc)RNA, which activates transcription at the interferons (IFNs) -γ locus. The lincRNA- Cyclooxygenase-2 (Cox2), notably expressed in Lipopolysaccharides (LPS)-stimulated dendritic cells, modulates immune gene expression by forming complexes with nuclear RNA-binding proteins (Duval et al., 2017; Yang F. et al., 2022).

Certain Lactobacillus-derived exosomes have shown promising anti-tumor and anti-viral properties, potentially reducing the occurrence of tumors and viral infections. *Lactobacillus gasseri* and *Lactobacillus jensenii* have been identified as particularly effective in stimulating the production of IFN-γ by human mononuclear cells from peripheral blood (PBMCs) (Jounai et al., 2018; Tsuji et al., 2018; Nicolò et al., 2021).

In summary, exosomes from lactic acid bacteria have a wide range of immunomodulatory effects, enhancing intestinal immune responses and reducing intestinal inflammatory and allergic reactions. Future research should explore the mechanism of Lactobacillus-derived exosomes in immunomodulation and develop their application in the prevention and treatment of immune-related diseases.

### Intestinal protection and microecological balance

Lactobacilli exosomes have emerged as a significant probiotic metabolite that plays a pivotal role in maintaining intestinal protection and microecological balance (Figure 1).

Exosomes act through various mechanisms to maintain the intestinal microecological balance, regulate the intestinal microbial composition, and uphold the stability of the intestinal microenvironment. For instance, certain proteins in *Lacticaseibacillus rhamnosus* GG-derived exosomes can competitively bind to the intestinal surface, reducing the adhesion of harmful bacteria and ameliorating the intestinal inflammatory response (Doron et al., 2005). Additionally, a highly expressed sequence (sRNA71) was identified in Exosomes which were isolated from the culture supernatant of *L. plantarum* WCFS1 using ultracentrifugation. sRNA71 substantially reduced Tp53 expression in HEK293T cells and suppressed the gene expression through binding to the 3′UTR of Tp53 mRNA (Yu et al., 2022). These exosomes contain bioactive substances that regulate the intestinal microbiome, such as extracellular enzymes, antibiotics, antimicrobials, antibiotic enzymes, iron carrier proteins, β-glucan, siRNA and polysaccharides, which can impact the type and quantity of intestinal flora, improve the growth and metabolism of probiotics in the intestine, and maintain intestinal health (Uddin et al., 2020; Díaz-Garrido et al., 2021; Yang Z. et al., 2022). Lactobacilli exosomes can also protect the intestinal tract by regulating intestinal immune function. Studies have demonstrated that *Lacticaseibacillus rhamnosus* GG-derived exosomes can enhance the integrity of the intestinal mucosal barrier, regulate the activity of immune cells in the intestine, and improve the intestinal immune function and disease resistance (Tong et al., 2021). Additionally, specific components of *Lacticaseibacillus rhamnosus* GG-derived exosomes (such as proteins, polysaccharides, etc.) also can interact with intestinal epithelial cells and regulate their signal transduction pathways, thereby affecting the integrity of the intestinal mucosal barrier and the balance of the immune response (Tong et al., 2021).

Recent studies using C57BL/6J mice have shown that *Lactobacillus plantarum* Q7 extracellular vesicles (Q7-EVs) enhance intestinal mucosal barrier functions and reduce inflammation and allergic responses. Q7-EVs were effective in alleviating DSS-induced colitis symptoms, such as colon shortening, bleeding, and weight loss, and decreased histological damage. They downregulated proinflammatory cytokines (IL-6, IL-1β, IL-2, TNF-α) and corrected gut microbiota dysbiosis, increasing anti-inflammatory Bifidobacteria and Muribaculaceae while reducing Proteobacteria, demonstrating their potential in improving gut health maintenance (Hao et al., 2021).

Some studies have also shown that Lactobacillus-derived exosomes. Influence the regulation of gut microbial composition and metabolites, promoting the growth of probiotic bacteria and inhibiting the growth of harmful bacteria. For example, *Lacticaseibacillus rhamnosus* GG (LGG) derived EVs could potentially alleviate intestinal inflammation by diminishing the activation of the Toll-Like Receptor (TLR4)- Nuclear Factor Kappa (NF-Κ) B1- Nucleotide-binding oligomerization domain (NLRP3) axis. The effectiveness of this treatment is evident in its ability to decrease pro-inflammatory cytokines such as TNF-α, IL-1β, IL-6, and IL-2. Furthermore, 16S rRNA sequencing reveals that LGG-EVs administration can alter the composition of gut microbiota in mice affected by colitis, subsequently influencing the microbiota’s metabolic processes (Tong et al., 2021). This process helps to regulate the intestinal microbial composition and promote the balance of the immune system. Additionally, exosome components have specific immunomodulatory functions, with some proteins inducing the proliferation and differentiation of immune cells and nucleic acid molecules enhancing the function of the intestinal barrier by binding to Toll-like receptors in intestinal epithelial cells (Caputi and Giron, 2018).

On the other hand, Lactobacillus-derived exosomes can also regulate the intestinal immune system. Extracellular vesicles derived from *Latocseibacillus. paracasei* (LpEVs) were effective in diminishing the levels of pro-inflammatory cytokines such as IL-1α, IL-1β, IL-2, and TNFα, which were initially elevated due to LPS stimulation. These vesicles enhanced the levels of anti-inflammatory cytokines IL-10 and TGFβ. In HT29 cells, LpEVs mitigated LPS-induced inflammation and reduced the activation of inflammation-related proteins like COX-2, iNOS, and NFκB, as well as the production of nitric oxide (Choi et al., 2020). Moreover, the polysaccharides and lipids in *Lactiplantibacillus plantarum* BGAN8 -derived exosomes can regulate the inflammatory response of the intestinal immune system, ameliorate intestinal inflammation and autoimmune response, and thereby maintain intestinal immune homeostasis (Bajic et al., 2020).

Recent research has demonstrated that exosomes from *Limosiactobacillus reuteri* strains DSM 17938 and BG-R46 promote the proliferation and differentiation of T cells and stimulate peripheral blood mononuclear cells (PBMC) and intestinal macrophages to release inflammatory cytokines, notably IL-6 and IL-1β. Additionally, they exert a modulatory effect by inhibiting the secretion of IFN-γ and reducing the secretion of TNF-α, which are typically induced by *Staphylococcus aureus* (Pang et al., 2022).

In summary, significant advances have been made in the study of Lactobacilli exosomes’ role in intestinal protection and flora homeostasis. Further research is warranted to explore the composition, structure, and function of Lactobacillus-derived exosomes and their mechanisms of interaction with intestinal microecological homeostasis. These findings hold great promise in the development of novel intestinal health products and therapeutic approaches.

## Isolation and purification of Lactobacilli exosomes: methods and techniques

The isolation and purification of Lactobacillus-derived exosomes have become a subject of great interest to researchers due to their potential applications in various fields. Due to the similarities in characteristics between Lactobacillus-derived exosomes and exosomes from other sources, the methods for isolation and purification should also be similar. Over the years, several methods have been developed for the isolation and purification of these exosomes, including ultrafiltration, gel filtration, polyethylene glycol (PEG) precipitation, isoelectric focusing, affinity chromatography, calcium ion column chromatography, and counter-current chromatography.

Ultrafiltration is one of the most commonly used methods to isolate Lactobacillus-derived exosomes (Lee et al., 2018; Wang et al., 2021). This method involves screening exosomes from the culture medium, and the separation and enrichment of exosomes can be achieved by varying the pore size and pressure of the ultrafiltration membrane. However, this method may result in the loss of exosomes and protein contamination during the separation process, necessitating a subsequent purification step.

Centrifugation (Lei et al., 2021; Kim et al., 2022) is another frequently used method to isolate exosomes. It involves separating exosomes from other cellular components, such as bacteriophages and cell walls, by centrifuging the culture medium several times. Although this method is simpler than the ultrafiltration method, the separation efficiency is relatively low.

Precipitation is another effective method for isolating Lactobacillus-derived exosomes, typically achieved by adding salts or acidic precipitants. It enables the quick and efficient separation of exosomes but is prone to exosome loss and protein contamination problems.

Chromatography (Baranyai et al., 2015; Sidhom et al., 2020) is a highly accurate method of separation and purification, which includes gel filtration chromatography, ion exchange chromatography, reverse phase chromatography, and many other techniques. Reverse-phase high-performance liquid chromatography (RP-HPLC) is widely used for the purification of exosomes. This method can improve purity without losing exosome activity and can obtain high-purity exosomes in a single operation in a single pass.

Affinity chromatography (Burkova et al., 2018; Sedykh et al., 2022) is a molecular recognition-based separation method that involves affinity chromatography and immunoaffinity chromatography, among others. These techniques use the binding of specific compounds or antibodies to exosome molecules to isolate exosomes, enabling efficient separation and purification. However, they require high-cost molecular recognition materials and specialized operational skills and are therefore currently less frequently employed for the isolation of Lactobacillus-derived exosomes.

The aggregate method is commonly employed for exosome extraction from lactic acid bacteria, which leverages the interaction force between aggregates and polysaccharides or proteins to isolate exosomes. Aggregating agents such as PEG (Weng et al., 2016) or polyacrylamide (PAA) (Li et al., 2023b) can be used to separate exosomes. PEG6000 is typically employed due to its optimal separation efficiency, as molecular weight greater than 6,000 diminishes its effectiveness. Meanwhile, PAA is effective in precipitating microbial cell walls and intracellular material together with exosomes. The aggregate method is a cost-effective, easy-to-control, and scalable technique; however, contamination of the target material is a concern.

Recently, some novel techniques have been proposed by researchers for the isolation and purification of exosomes, such as magnetic-bead-affinity chromatography (MBAC) (Wu et al., 2021). MBAC enables the quick and efficient separation and purification of exosomes from complex samples by selecting antibodies that specifically bind to target exosomes.

Each of these methods has its advantages and limitations and can be selected based on the nature of the desired exosome and the research objectives. For instance, ultrafiltration and gel filtration are commonly employed to isolate exosomes with different molecular weights, while PEG precipitation is useful for the large-scale preparation of exosomes. Isoelectric focusing is an effective method for purifying exosomes with electric charges, and affinity chromatography can be used to purify exosomes with specific activities by selecting appropriate affinity substrates.

Moreover, operational steps are often more intricate. Other factors that can affect the extraction efficiency include culture medium, culture conditions, and time. A medium rich in carbon and nitrogen sources and the addition of certain small molecules such as citric acid can improve exosome yield and isolation efficiency (Royo et al., 2016).

The selection of a method depends on the exosomes’ size, morphology, composition, and intended use. In particular, ultrafiltration and molecular sieve filtration are commonly used techniques. The former allows for the screening of particles of desired size through different pore sizes, while the latter utilizes molecular sieve materials’ effect to filter by molecular size. While these methods have been useful, more research is needed to explore exosomes’ composition and biological functions.

## Application of Lactobacillus-derived exosomes

Lactobacillus-derived exosomes have been investigated for their therapeutic effects on a range of diseases. For instance, they have been shown to have anti-inflammatory and immunomodulatory effects, making them attractive candidates for treating autoimmune and inflammatory diseases. Due to the potential presence of heterologous proteins in Lactobacillus-derived exosomes, oral administration of Lactobacillus-derived exosomes is an ideal delivery route. Studies have demonstrated the feasibility of using exosomes as a therapeutic strategy via oral administration to treat diseases. In a study on a mouse model of colitis, bovine colostrum-derived exosomes administrated orally were found to significantly alleviate colonic inflammation and promote tissue repair (Han et al., 2022; Li et al., 2023a). Furthermore, food-derived exosomes have been shown to improve gut microbiota dysbiosis and intestinal barrier function, which are associated with a range of diseases such as human irritable bowel syndrome and inflammatory bowel disease (Díez-Sainz et al., 2021a). These findings suggest that Lactobacillus-derived exosomes may have potential therapeutic applications in various disease settings.

Lactobacillus-derived exosomes have been shown to contain various functional molecules, such as proteins and RNA, which can influence the composition and function of the gut microbiota. For example, a recent study demonstrated that exosomes could modulate the gut microbiota of mice by promoting the growth of beneficial bacteria and inhibiting the growth of harmful bacteria, which could potentially improve gut health in humans (Díez-Sainz et al., 2021b). Furthermore, Lactobacillus-derived exosomes have been shown to regulate the gut-brain axis, which is a bidirectional communication system between the gut and the central nervous system and is involved in the regulation of a range of physiological functions such as mood and appetite in humans (Evrensel and Ceylan, 2015). These findings suggest that Lactobacillus-derived exosomes may be useful for modulating the gut microbiome and the gut-brain axis, which could have implications for a range of health conditions.

Despite the potential of Lactobacillus-derived exosomes, there are still some challenges that need to be addressed. For example, the isolation and purification of Lactobacillus-derived exosomes are not yet fully optimized, which could affect their yield and purity. Furthermore, the mechanisms of action of Lactobacillus-derived exosomes *in vivo* are not fully understood, and their long-term safety needs to be further investigated.

In conclusion, Lactobacillus-derived exosomes represent a promising area of research in the field of medicine, with potential applications in drug delivery, disease treatment, and gut microbiome modulation. The latest research findings have demonstrated their effectiveness in promoting tissue repair, modulating the gut microbiota, and regulating the gut-brain axis. While further research is needed to fully understand their potential applications and address remaining challenges, the progress in the field of Lactobacillus-derived exosomes has provided new insights and potential treatments for a range of diseases.

## Engineering Lactobacilli exosomes for enhanced functionality

The engineering of Lactobacilli exosomes involves the construction of exosomes with specific functions using gene editing and transformation techniques. There are two main aspects of engineering Lactobacilli exosomes: modifying the bacteria to produce more or more effective exosomes, and genetically or chemically modifying the already isolated exosomes to give them new or improved functions. Researchers have employed recombinant genetic engineering techniques to insert exosome-related genes into the genome of cells to improve exosome production (Jafari et al., 2020). They have also optimized the culture conditions of lactic acid bacteria to enhance exosome production (Garcia et al., 2015). To modify exosomes, researchers have used gene editing techniques to implant specific peptide sequences on the surface of exosomes to give them specific recognition and binding ability (Ye et al., 2020). Chemical modification methods have also been employed to add molecules such as polysaccharides and peptides to enhance their biological activity (Luo et al., 2021; Yang Q. et al., 2022).

In addition, exosome engineering can be applied to the preparation of novel vaccines. For example, researchers integrated the human papillomavirus E7 protein gene into the DNA sequence of *L. lactis*, resulting in a better immune-protective effect in mouse experiments (Smalley Rumfield et al., 2020; Krishnan et al., 2022), which implies the potency of Lactobacilli exosomes as the vaccine carrier.

In conclusion, while the engineering study of Lactobacillus-derived exosomes is still in its early stages, it has enormous potential for various applications. Further research is needed to refine the feasibility and application prospects of this technology.

## Conclusion and perspectives

Lactobacillus-derived exosomes, a novel type of biological drug, hold immense potential in the medical realm. They also modulate intestinal microflora, thereby maintaining gut health and boosting immunity (Figure 1). These exosomes, acting as natural carriers, can transport diverse drug molecules with excellent therapeutic benefits and mitigate adverse drug reactions. However, despite these advantages, lactobacillus exosome research and application are still challenging. Firstly, the isolation and purification methods employed for these exosomes are not yet perfect and require optimization to enhance their yield and purity. Secondly, the intricate and heterogeneous structure and composition of Lactobacillus-derived exosomes necessitate a more comprehensive analysis of their structural and functional relationships to unravel their mechanism of action in living organisms. Furthermore, additional investigations are imperative to evaluate drug delivery mechanisms, *in vivo* distribution, metabolism, and long-term safety of Lactobacillus-derived exosomes. In conclusion, while lactobacillus exosome research offers a promising avenue for drug delivery, a significant number of basic and clinical studies are imperative to realize their potential in the pharmaceutical sector. Moreover, standardized production and quality control protocols for Lactobacillus-derived exosomes need to be strengthened to ensure their safe and efficacious application.

## Author contributions

RL: Conceptualization, Formal analysis, Investigation, Writing – original draft, Writing – review & editing.
